# On the Routing Protocol Influence on the Resilience of Wireless Sensor Networks to Jamming Attacks

**DOI:** 10.3390/s150407619

**Published:** 2015-03-27

**Authors:** Carolina Del-Valle-Soto, Carlos Mex-Perera, Raul Monroy, Juan Arturo Nolazco-Flores

**Affiliations:** 1 Department of Electrical and Computer Engineering, Tecnológico de Monterrey, Ave. Eugenio Garza Sada #2501 Sur, Monterrey, NL 64849, Mexico; E-Mails: carlosmex@itesm.mx (C.M.-P.); jnolazco@itesm.mx (J.A.N.-F.); 2 School of Science and Engineering, Tecnológico de Monterrey, Campus Estado de México, Carretera al lago de Guadalupe Km 3.5, Col. Margarita M. de Juárez, Atizapán 52926, Mexico; E-Mail: raulm@itesm.mx

**Keywords:** wireless sensor networks, jamming, network resilience, routing protocols, network performance

## Abstract

In this work, we compare a recently proposed routing protocol, the multi-parent hierarchical (MPH) protocol, with two well-known protocols, the *ad hoc* on-demand distance vector (AODV) and dynamic source routing (DSR). For this purpose, we have developed a simulator, which faithfully reifies the workings of a given protocol, considering a fixed, reconfigurable *ad hoc* network given by the number and location of participants, and general network conditions. We consider a scenario that can be found in a large number of wireless sensor network applications, a single sink node that collects all of the information generated by the sensors. The metrics used to compare the protocols were the number of packet retransmissions, carrier sense multiple access (CSMA) inner loop retries, the number of nodes answering the queries from the coordinator (sink) node and the energy consumption. We tested the network under ordinary (without attacks) conditions (and combinations thereof) and when it is subject to different types of jamming attacks (in particular, random and reactive jamming attacks), considering several positions for the jammer. Our results report that MPH has a greater ability to tolerate such attacks than DSR and AODV, since it minimizes and encapsulates the network segment under attack. The self-configuring capabilities of MPH derived from a combination of a proactive routes update, on a periodic-time basis, and a reactive behavior provide higher resilience while offering a better performance (overhead and energy consumption) than AODV and DSR, as shown in our simulation results.

## 1. Introduction

Wireless sensor networks (WSNs), sometimes covering wide geographical areas, are being deployed for a large number of applications, as they have become one of the most suitable technologies for supporting services, such as air quality monitoring, vehicular traffic monitoring, street lighting optimization, smart parking, among others [1]. In these scenarios, WSNs are exposed to time variant conditions, such as changes in the propagation of radio signals, temperature, humidity, wind, interferences and deterioration of the physical components. This random behavior affects even those WSN applications where nodes are static. Thus, the network can face from favorable to adverse conditions, and yet, it must operate uninterruptedly with the best possible performance, providing continuous information services.

In addition, other factors can impact the services delivered by the networks, such as faults and attacks. For faults, intermittent or dropped radio links can change the network connectivity. For attacks, and in particular if nodes are static, the exposure to threats can be increased, since evasion tactics are not possible [2]. Therefore, an important consideration in the design of sensor networks is the notion of resilience, where the routing protocol plays a key role in the self-configuring capabilities of the WSN to deal with adverse scenarios.

Among different types of menaces, jamming, an intentionally generated interference to degrade the services offered by a WSN, is one of the most feasible kinds of attacks. Cryptographic mechanisms cannot prevent jamming from affecting a WSN, since the interference can impact on the transmission and reception of legitimate packets. Therefore, an analysis of the effects of jamming can be useful as a way to evaluate the performance of WSNs.

Some of the parameters studied to identify the jamming nature are: resource limitations, wireless communication medium, the mobility of nodes and absence of infrastructure [3]. Another performance metric is the amount of changes in the radio links (new or dropped links) implying node connections or disconnections and, hence, a constant change on WSN topology (thus, this metric is related to network connectivity). Ultimately, high changes on the availability of routes means that the routing protocol should be able to react quickly to any changes and update routes efficiently. Additionally, when a network is disturbed, node time recovery ought to be measured [3]. Nodes' forwarding packet capability is also essential in a WSN and can be significantly affected by the characteristics of the wireless medium and noise. Nodes that carry a high amount of traffic are likely to exhaust their energy faster, and this causes network failures or traffic spikes generating packet loss. Moreover, the addition of new nodes to the network also causes changes in routing tables and topology [3]. However, it is very important to analyze the whole scenario, because sometimes, node failures or poor connectivity can have similar effects as a jamming attack [4].

### 1.1. Objective

The purpose of this paper is to study the effect of several types of jamming attacks (in particular, random and reactive) in a WSN. More specifically, we shall study how network performance degrades, when subject to jamming and when using any of the following routing protocols: *ad hoc* on-demand distance vector (AODV), dynamic source routing (DSR) and the multi-parent hierarchical (MPH) protocol [5,6]. Network, MAC and energy performance are captured in terms of several proposed metrics.

Throughout our research, we have found both that MPH is more resilient to jamming attacks, as well as to adverse conditions of the wireless environment in that it offers a better network performance (overhead and energy consumption) than DSR and AODV. This is both because MPH encapsulates the attack to a smaller network segment better and because of the self-configuring capabilities of MPH, derived from a combination of a periodic and proactive route update. Thus, at least for certain scenarios, there is room for using alternate protocols to AODV or DSR. Our results ultimately motivate further research in studying other network metrics that enable, in the context of MPH, a timely detection of jamming and other kinds of attacks.

We describe the organization of the rest of the paper. In Section 2, we present related work. Section 3 reviews jamming attacks. Section 4 discusses protocols, jamming and metrics analysis. Section 5 shows the description of simulator parameters and network architecture. Section 6 analyzes the results and discussion. Section 7 explains different spatial node distributions. Finally, conclusions are given in Section 8.

## 2. Related Work

Kiwior *et al.* [7] study mobile *ad hoc* networks (MANETs); in particular, link quality due to jamming, interference, coverage range or other generic characteristics of the communication medium in an airborne environment. They analyze the connectivity and performance of known protocols using metrics, such as the packet delivery ratio (PDR), end-to-end delay and other overhead metrics.

Mingyan *et al.* [8] provide optimal detection tests that show what knowledge of a network an attacker node may acquire. The authors analyze the difficulty of detecting an attacker and the collective impact of defending a network. The authors take into account the probability of jamming and the transmission range. The work of Misra *et al.* [9], by contrast, studies a metric for measuring the degree of jamming affecting each node, providing the jammer's localization centrally. Furthermore, the authors complement the method with a decentralized setting, where each node measures the perceived jammer power in order to observe the degree of damage based on coordinates. According to the parts of the network affected by the jamming, a zoning map of the jammed area is created, as well as measures for decreasing node energy consumption are established. Likewise, the work cited in [10] studied a local perspective for detecting jamming. The metric used is throughput. The authors make a comparison between normal levels of throughput and interference levels of throughput (or abnormal). The authors establish two categories of jamming: signal strength-based and protocol behavior-based. Moreover, they try to detect the jamming affected zone (through PDR and throughput) and locate unaffected nodes to isolate them from the attack. This technique favors network performance and the study of the self-configuration feature the of routing protocol.

Xu *et al.* [4] examine jamming attacks and the corresponding defense strategies. They also analyze different methods for detecting the presence of a jammer node. The authors characterize and then analyze different types of jamming presented in a wireless medium, namely: constant, deceptive, random (which, as shall be discussed below, is a little different from our definition of random jamming) and reactive. To detect the presence of jamming in either the transmitter or receiver, the authors suggest using a number of measures, particularly signal strength, carrier sensing time, the packet send ratio (PSR) and the packet delivery ratio (PDR).

Wireless mesh networks are studied by Jiang and Xue [11]. There, the authors analyze the benefits of dynamic channel assignment, alternating routes and channels and effective routing when a network defends against a jamming attack. They also study the effect of restoration and how it affects the latency and throughput of the network. By way of comparison, in [12], Awerbuch *et al.* propose a MAC protocol capable of defending a network against a jamming attack at the physical layer. There, a model that takes into account the difference between a jamming attack or a collision is proposed, and the energy efficiency of the protocol on different attacks is shown.

By contrast, in [13], the authors analyze how powerful an attacker can become with respect to energy consumption, transmission range and coverage. They point out that the objectives of a jammer node include obstructing internode communication (especially with the collector node) and increase the energy consumption of the network node. One of the biggest challenges an attacker must face is to know the MAC protocol that the nodes run, so as to determine how one should attempt to disrupt communication.

The aforementioned works consider a number of input variables for the jamming detection mechanisms; *i.e.*, packet delivery ratio, signal strength, carrier sense metrics, *etc*. Therefore, some of the methods presented there may result in being more effective than others depending on the conditions of the attack and the scenarios. One of the motivations for this work was to conduct a comprehensive analysis on the metrics that can present changes under a jamming attack. The results of this research could be used later to create better detection methods by choosing suitable input variables. However, for a better understanding of the analysis, the underlying routing protocol should be considered as a factor that influences the behavior of the metrics. Some of the most prominent routing protocols for WSN are the AODV and DSR; since these protocols have different inner mechanisms, then the behavior of the measured variables would be different for each of them; thus, a methodology that includes a larger number of metrics than previous works and, at the same time, the influence of the routing protocol would extend some results of the state-of-the-art. Besides, the recently proposed protocol, MPH, which has design principles to deal with adverse conditions of the wireless environment, is also considered in this paper as a rival of AODV and DSR for certain scenarios.

## 3. Jamming Attack Models

A jammer is defined in [14] as “…an entity who is purposefully trying to interfere with the physical transmission and reception of wireless communications”. There is a number of models for jamming, and some of them will require more resources than others when they are carried out in a real scenario. Budget, effectiveness, technical complexity and energy consumption are some of the factors that are considered when defining an attack strategy.

In constant jamming, for instance, a constant emission of a radio signal is used to block a node from receiving signals from others. In addition, if that target node MAC layer is based on a carrier sense scheme, then it will not be able to transmit either, since the medium channel will always be busy. This kind of jamming produces an effective denial of service (DoS). Yet, it may require purchasing specialized hardware, such as a waveform generator or a power amplifier, since, often, a power supply or a battery with enough capacity is needed in order to provide the energy demanded by the jamming equipment. This makes jamming deployment less practical, but more importantly, it eases the identification of a suspicious device. Indeed, when a DoS becomes so evident, constant jamming can be easily detected; therefore, it might not be a reasonable long-term attack strategy.

In the process of defining how a jamming attack should be attempted, one hence may consider special hardware or even a special configuration of a wireless node. However, from the attacker's point of view, for the sake of both simplicity and inexpensiveness, the hardware that can best interfere with the channel frequencies of the target node is one that is originally intended to serve as a legitimate node of the WSN, and since, in most cases, such a device is low-cost, the attack can be very practical. In our work, we shall consider a practical scenario where the jamming is produced by a device with the same MAC and physical layer as the rest of the nodes of the WSN. Thus, the packets produced by the jamming device will have the same structure as the packets generated by legitimate nodes.

In the comparative development of this work, we have used two strategies for jamming: random and reactive. The rationale behind this selection decision is as follows. Random jamming is, on the one hand, one of the simplest types of jamming to implement and fairly difficult to detect. On the other hand, reactive jamming is both complex to implement, but rather difficult to detect. We introduce our jamming models below.

### 3.1. Random Jamming

We are using a random jamming model where packets are produced following a uniform distribution and *ρ* is the number of packets per second generated by the jammer node. As expected, *ρ* can be adjusted to increase or reduce the level of the jammer aggressiveness. A low value of *ρ* indicates a reduced interference activity, and as consequence, the DoS will not be constantly carried out. However, from a detection jamming mechanism point of view, it would be quite difficult to detect this attack, since its effects can be misinterpreted as normal interference due to the wireless environment (other wireless networks and devices sharing the same bandwidth). By way of comparison, a high value of *ρ* will produce a more severe DoS effect, and it could be evident that an abnormal interference activity is being performed; this scenario can be considered as a worst case for the WSN in terms of performance degradation.

We note in passing how our random jamming model compares to that of [4]. If we set *ρ* to a very large value, we end up with a model that Xu *et al.* call deceptive, which constantly injects regular packets into the channel (without any gap between subsequent packet transmissions). By contrast, if we set *ρ* to an intermediate value, then we end up with a model very similar to what they call random, the only difference being that, instead of sending a burst of two or more packets, we send only one. Our packet inter-generation time corresponds to the pause step of Xu *et al.*, where the node falls asleep.

### 3.2. Reactive Jamming

As in [4], reactive jamming is an attack strategy where the jammer will emit a packet with the objective of colliding with a legitimate packet. The jamming device keeps monitoring the wireless medium; once it detects a packet, it then generates a collision one immediately after. Note that the attack will be successful if at least one bit of the legitimate packet is altered, and there is no error correction scheme, only an error detection mechanism. This attack uses less energy than the random jamming strategy.

In this paper, we use rates from 50 packets/s to 80 packets/s in order to observe the network behavior under the simplest attack and that is easy to detect by the neighboring nodes, under measurements of signal strength and collision rates. On the other hand, in the other extreme, one of the most difficult attacks to detect is reactive jamming. This is because the attacker node is observing the channel in order to listen to an activity, and it causes collisions.

Table 1 presents the main characteristics and assumptions considered in this work on the implementation of two known types of jamming: random and reactive. With this table, we intend to justify the use of each of these types of jamming in order to compare the behavior of the network under each one. In addition, we will study these types of jamming by a fair comparison of two known routing protocols, AODV and DSR, and the proposed MPH, as explained earlier in [6] and evaluated under general performance metrics in [5].

Other jamming attacks are more sophisticated. Intelligent jamming is aware of the MAC or network protocols, and they can interfere with specific packets in order to maximize the jamming gain. We shall not consider this type of jamming, since we would like to focus on general jamming strategies, which can be applied despite the underlying routing and MAC mechanisms.

## 4. The Role of Routing Protocols to Resilience in Self-Configuring WSNs

In WSNs, resilience capabilities can be provided by the self-configuring and adaptation mechanisms, for instance the power transmission of the nodes can be adjusted dynamically, the MAC algorithm may be aware of the wireless medium in order to optimize the performance and the routing protocol can change the topology according to the state and availability of the radio links, energy consumption, traffic balance, quality of service and other criteria. For some well-known routing protocols for WSNs, self-configuring is inherent to the design; reactive protocols, such as AODV [15] and DSR [16] update the routes when they are required; while in the MPH routing protocol [6], nodes update the routes on a periodic-time basis and when changes in the neighborhood have been detected. Despite the differences among the three aforementioned routing protocols, they use self-configuring mechanisms to build and update the topology when the conditions of the wireless environment and the available resources change. As a result, resilience to faults and attacks can be intrinsic to the above protocols. In this section, we will explain the fundamentals of the above protocols and present a brief example of their behavior under a jamming attack.

### 4.1. Fundamentals of AODV, DSR and MPH Routing Protocols

In AODV, a node sends an RREQ packet (route request) in broadcast mode in order to apply for a route. If a node receives this packet, it first checks if it has previously received a record of this RREQ packet. If the packet is not registered, the node retransmits it again, increasing the number of hops and creating a reverse path (the path to the node that this RREQ has reached). A node that has received an RREQ packet can only respond with an RREP packet (route reply) to confirm a route in only two cases: if the node is already the destination or if the node has an available route to the destination. This procedure continues until the source is reached. The source node may receive RREP packets from different nodes as a confirmation of various possible routes to the destination. In this case, the source node has two criteria for selecting the best route: the route that has the least number of hops or the route that has the highest sequence number. Then, nodes can have multiple routes to the same destination; the node generally chooses the shortest route.

In DSR, when a source node has a packet to send to a destination, it first looks in its cache to see if it has a route to that destination; if so, it creates a new packet header containing the path with the necessary hops to reach the destination and sends it. If there is no route to that destination in its cache, the node starts the route discovery mechanism, where the source node sends an RREQ broadcast packet, which contains the identifier of the source node and that of the destination node whose route is to be discovered; it also contains a unique identifier for the RREQ. Each intermediate node makes a copy of the received RREQ and adds its identifier. When a node receives a RREQ, it first looks in its cache to find out if it has the route to the destination node. If so, the node responds with an RREP to the source instead of forwarding the RREQ. In that RREP, the node writes all nodes that the received RREQ has passed through and the nodes of the route found in its cache. If the node does not find a route in its cache, it adds its address in the packet and forwards it via broadcast. Once the source node receives the RREP, it stores the route in its cache. This route will be included in the header of each subsequent packet that the node sends, so that all nodes that receive the packet will know to which next node they should resend. As with AODV, nodes can have multiple routes to a destination and choose the lowest hop count.

MPH is a routing protocol that creates a logical hierarchical network topology where the node hierarchy is given by its location level in the hierarchy (the lower the level, the lower the hierarchy) [6]; basically, the root hierarchy (coordinator node) will have the highest hierarchy. When a node is in a specific hierarchical level (or generation), the nodes that are connected to it and have a higher hierarchical level are parent nodes. On the other hand, nodes that are connected to any parent node and that have lower hierarchical level are child nodes. The hierarchical topology will be formed with routes that minimize the number of hops from a given node to the coordinator; this restriction will reduce the overall energy consumption of the network. The MPH routing protocol allows a child node to have one or more parents. As a result, a node can share both children and parents with another node belonging to the same hierarchical level or generation.

When a node cannot reach a specific neighbor node, then it deletes it from its neighbor table. This is a very important feature that helps to deal with the dynamic conditions of the wireless environment, such as loss of links, damaged nodes, battery exhaustion, *etc*. Besides, a proper neighbor update is especially important during a jamming attack, because this action has the effect of isolating the affected area and modifying the network topology, for instance re-routing the traffic using the rest of the network; this is true even if the attacker keeps moving. Moreover, since in the MPH protocol, the neighbor discovery packets are transmitted periodically, when the jamming attack finishes, the node can be discovered again, and it starts to behave as a normal node. This feature, intrinsic to MPH, is a proactive way to update the neighbor tables and provides to the network some resilience capabilities; this is not the case for other routing protocols, such as AODV and DSR: they may need special countermeasures against jamming attacks. In MPH, in order to assign a score to the steadiness of a neighbor, a persistence variable was defined, which is related to the number of times a neighbor node responds to the discovery packets; when a node replies to the discovery packet, it receives a maximum persistence value, and for each time the neighbor does not respond, then it will be decreased by one. If the persistence variable reaches zero, then it is concluded that the node is not responding, and it is deleted from the neighbor table.

The MPH protocol mainly uses three packets: the *ND* (network discovery), the *NDR* (neighbor discovery response) and *NDRACK* (neighbor discovery response ACK) packets. At initial state, if the node is the coordinator, then we set its hierarchy to the maximum value; otherwise, we simply set it to zero. Then, each node sends periodically *ND* packets to initiate the discovering and updating of its neighbors. When a node receives an *ND* packet, then it answers with an *NDR* packet, which includes its hierarchy information. The node that receives an *NDR* packet will reply with an *NDRACK* acknowledgment packet that also includes its hierarchy level. When an *NDR* or *NDRACK* packet arrives, the updating of the hierarchy for a node except the coordinator is summarized as shown below:
1. Receive packet from neighbor .2. Update neighbor table .3. { If } *neighbor_hierarchy* > *this_node_hierarchy* + 1 : a. Set the neighbor as the parent (next node in the neighbor table); b. It assigns itself a hierarchy level smaller than its parent : *this_node_hierarchy* = *neighbor_hierarchy* − 1 . c. Send a *ND* packet to advertise its hierarchy in the neighborhood .4. If *neighbor_hierarchy* == *this_node_hierarchy* + 1 ,{ then } set the neighbor as another parent .

When a node has data to transmit, then it creates an MPH packet and sends it to a parent node; if the parent node that receives the packet is the coordinator, then the packet has reached its destination; otherwise, the packet will be forwarded to any of the parent nodes.

**Figure 1:**
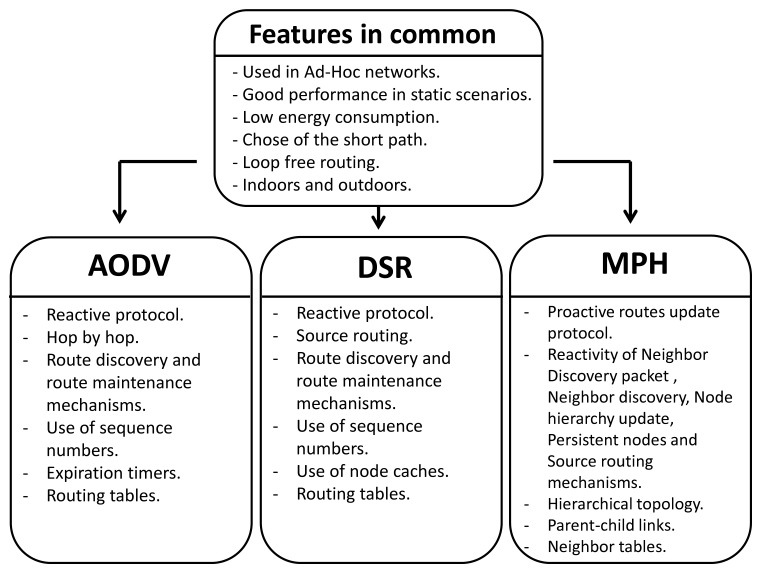
Important features of the *ad hoc* on-demand distance vector (AODV), dynamic source routing (DSR) and multi-parent hierarchical (MPH) protocols.

Figure 1 shows the most important features of the AODV, DSR and MPH protocols.

### 4.2. Example of Self-Configuring Mechanisms in WSNs

**Figure 2:**
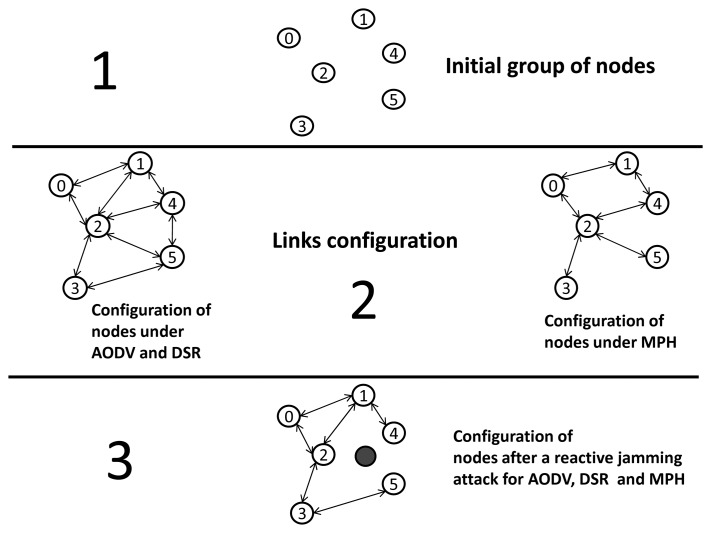
Self-configuring WSNs: AODV, DSR and MPH under a reactive jamming attack.

Figure 2 shows an example of the link configuration for a given WSN running the AODV, DSR and MPH protocols, this example consists of three phases: in the initial phase, the nodes are switching on; in the second phase, the network has been formed according to the underlying routing protocol; and in the third phase, we can see the resultant topology under a reactive jamming attack. In the second phase, each routing protocol establishes the links according to their rules. For AODV and DSR, links are established within the coverage radius of the nodes. Regarding MPH, parent-child links are set with a hierarchical topology with respect to a given coordinator node (node zero). The third stage presents how the topology for AODV, DSR and MPH is reconfigured after an attack caused by a jammer node represented by the shaded node. Let us assume that this node produces the reactive jamming (which is the worst case scenario for the WSN presented in this paper) and causes the links next to it to become unusable. The logical links are dropped due to the partial overlapping of the coverages of the jammer node with Nodes 2, 4 and 5 having in common the lost links. This situation is caused because the attacker node emits a packet when it listens to any packet transmission, causing collisions and corrupting the legitimate packet. The nodes under the MPH protocol update their neighbor tables as soon as they realize that there are no packets being received from some neighbors. In AODV and DSR, routing tables are also updated through error messages when the links are already dropped. The nodes in the WSN with AODV and DSR increase the average number of packet retransmissions and the number of MAC algorithm retries, because the jammer node constantly maintains the reactive attack. After the topology update, these links are lost, and error packets of these routes are received by the nodes. For MPH, nodes quickly realize the futility of these affected links due to persistence and reactivity mechanisms [6]. In addition, the periodicity of the neighbor tables' update produces these links that are discarded without sending extra packet notification. Finally, this reflects lower power consumption and better performance for a network under the MPH protocol.

## 5. Description of Simulator Parameters and Network Architecture

**Figure 3:**
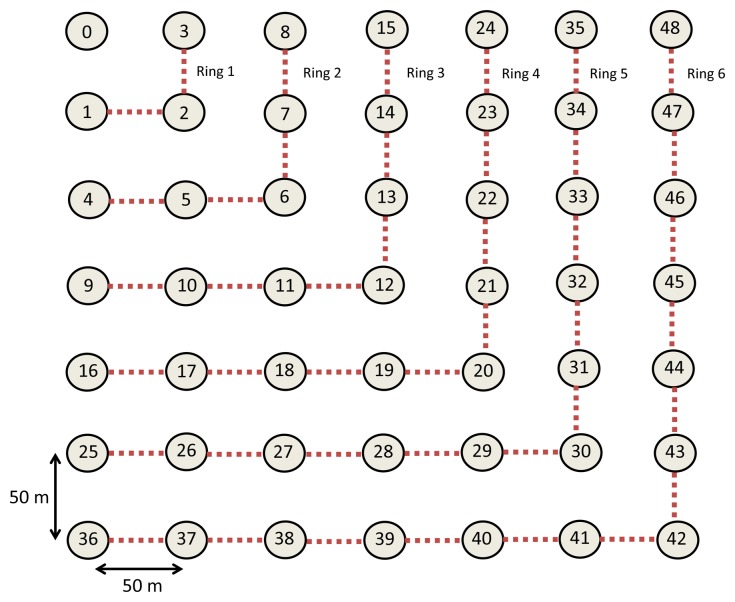
Network topology.

These experiments are based on a grid of 49 nodes, where 48 of them send data with a rate of 1%–4% packets/s to a coordinator node (in the upper-left corner) functioning as a sink node. The grid is shown in Figure 3. The network consists of a MAC layer under the CSMA/CA (Carrier Sense Multiple Access with Collision Avoidance) protocol, with a maximum of three retransmissions per packet and maximum of five CSMA retries, while the nodes are waiting for the channel to be idle before transmission. The packet rate of the jammer node varies according to the type of jamming attack; random (*ρ* = 50 packets/s), random (*ρ* = 80 packets/s) or reactive.

Let us consider using a transmission power of 0 dBm [17] for active transmission mode according to the CC2530 chip [18] and a receiving power of −85 dBm according to the IEEE standard 802.15.4 [19]. With these power values, we obtain an average coverage radio of 50 m for sensors following Equation (1) for outdoors [19]:

(1) $$d=8*10^{\frac{P_{t}-P_{r}-58.5}{33}}$$

where *d* is the coverage radio, *P_t_* is the transmission power in dB and *P_r_* is the receiving power in dB.

Table 2 shows the physical, MAC and network layer parameters that were used in the simulator.

### 5.1. Performance Metrics and Sampling Periods

The retransmissions metric is defined as the number of times to retransmit a packet because the source node does not receive an acknowledgment. We establish a maximum of three retransmissions for each packet; once this value has reached, the packet is dropped. This metric indicates the link state of the network and can provide insight connections and the connections of nodes. The lower this metric value, the better.

The CSMA retries metric is the number of times a node runs the inner loop of the CSMA/CA algorithm. When a node is about to transmit a packet, it first listens to the channel and notes if the channel is available for transmission (carrier sense). If the channel is busy, then the node waits a random delay for another carrier sense. This cycle is what we call CSMA retry. This parameter has a maximum default value of five times in the IEEE 802.15.4 standard [19]; once this number has been reached, the packet is discarded. This metric gives an indication of the wireless medium state that is influenced by the amount of packet collisions that force repeated CSMA backoffs. The lower this metric value, the better.

The tries metric is defined as the number of times the coordinator node needs to communicate with every single node in the network. This metric also is known as route availability. The coordinator is a robust node and has powerful processing capability to be aware of the entire network topology. We set a maximum of nine tries for each node. This metric relates connectivity to the link state and topology changes. The lower this metric value, the better.

For the metrics retransmissions and CSMA retries, we set a sampling period of 100 s, where for the first 30 s, the network is in normal mode. Then, in the next 30 s, a node jammer is introduced in any of the positions evaluated (near, medium or far from the node coordinator, which is always the destination node). Finally, for the next 40 s, the jammer node is removed, and the network returns to a normal state.

For the tries metric for connecting from the coordinator to each node, we established 100 s of normal network mode and 100 s in the presence of a jammer node. Then, we run this “ping” process for every single node of the network by the coordinator, so this constitutes approximately 20 repetitions of the process in a period of 100 s for significance in results. We insist that we have set the maximum tries to nine per node.

The achievable nodes by the coordinator metric is defined as the number of nodes that can answer the queries from the coordinator. Ideally, the coordinator must know the whole network topology and, therefore, should be able to reach all nodes when required. The higher this metric, the better for the network. It is important to note that this metric is related to the tries metric with each of the nodes. We have set a maximum of nine tries to reach a node.

Energy is a metric that quantifies the consumption of resources on each node. When a network has low performance, the energy consumption will be higher, because the number of collisions increases and, thus, retransmissions and CSMA retries. Furthermore, when the routing protocol is not able to recover quickly from an attack, the link state and energy consumption will be increased. For assessing this metric, we established 100 s of normal network mode and 100 s in the presence of a jammer node.

## 6. Results, Analysis and Discussion

Results of the studied variables are displayed by statistical graphs and 2D spatial distribution figures. Statistics help us to note how the most relevant information on nodes is organized. The spatial distribution gives us an idea of which nodes or node zones are affected and what the impact is in the whole network. Tables that accompany the following results summarize the information contained in the figures presented in this section and help to compare the results for each of the protocols.

### 6.1. Statistical Analysis of Performance Metrics

The methodology used to present the following results is the box and whisker method, which shows the distribution of data for each of the evaluated metrics and allows us to have a representation that describes the dispersion and symmetry characteristics. It represents three quartiles (*Q*_1_, *Q*_2_, *Q*_3_) and the minimum and maximum data values on an aligned rectangle, horizontally or vertically. *Q*_1_, the first quartile, is the value greater than 25% of the values of the distribution. *Q*_2_, the second quartile, is the median of the distribution; it is the value of the variable that takes center place in a set of ordered data. *Q*_3_, the third quartile, is the value that exceeds 75% of the values of the distribution. The interquartile range is *Q*_3_–*Q*_1_, and it represents 50% of the nodes. In order to develop this method, we followed the standard approach described in [20].

For the purpose of the results analysis, it is important to note that the graphs of the box and whisker method has been built with a whisker coefficient of 1.5. The extremes in each box represented by a line show the maximum and minimum values of observed data. The ends represented by X are the 1% at the bottom and the 99% at the top of the box. The filled squares shown in the boxes represent the mean of the data.

#### 6.1.1. Retransmissions with Box and Whiskers Representation

**Figure 4:**
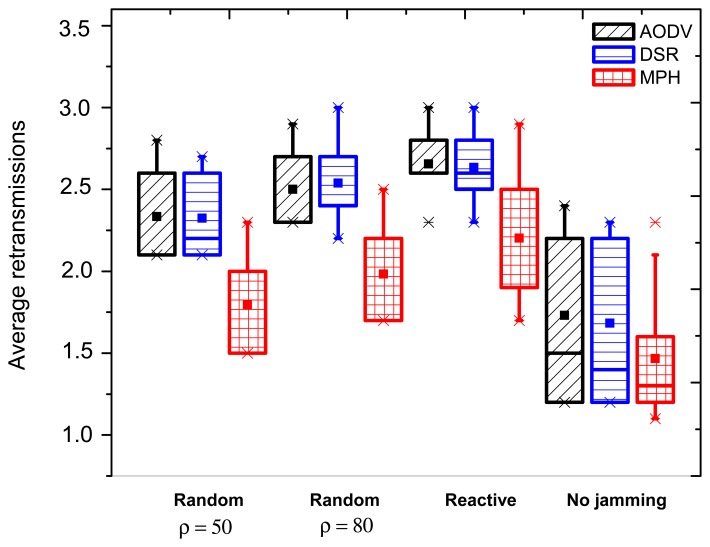
Average retransmissions when the jammer node is in the middle of the topology.

Figure 4 shows the average retransmissions for the AODV, DSR and MPH protocols for each of the attacks studied in this work; random (*ρ* = 50, 80 packets/s) and reactive. The figure presents the position when the jammer node is in the middle of the topology.

For the retransmissions metric with no jamming activity, the MPH protocol has a smaller box, which means that the values of average retransmissions are below the values that present the AODV and DSR protocols. Then, the MPH protocol has 15% less average retransmissions than AODV and 13% less than DSR. We observe that when there is no presence of a jammer node, the bottom of the box is smaller than the top. This means that average retransmissions values between 50% and 75% of the data population are more dispersed than data between 25% and 50%.

Figure 4 describes the three different attacks for each of the studied protocols with the jammer node in the middle of the network topology. Note that in the three jamming types, the boxes are short in size, so that we can infer that when the jammer node is in the middle of the topology, it affects more nodes, because it can cause an attack that spreads in the concentric circles form. However, boxes for MPH indicate that the WSN can be resistant to this wave of attack, because their values are significantly lower than AODV and DSR. With respect to the random jamming with *ρ* = 50 packets/s, MPH has average values of 23% lower than AODV and DSR. Regarding the random jamming with *ρ* = 80 packets/s, MPH shows an improvement of 20.5% over AODV and 21.7% over DSR. Under reactive jamming, MPH reacts 16.7% better than the other two studied protocols, and we note again that under the most aggressive network attack, the lowest number of nodes affected by the jamming is when the MPH is used.

In what follows, we explain the results for both cases, when the jammer node is near and far from the coordinator. For the near case, we obtained that over the network nodes, there are significant differences in the values of this metric. Moreover, the distance or proximity of the nodes with respect to the coordinator influences collisions, packet loss and, therefore, retransmissions. This is because the nodes that are closer to the coordinator node forward more traffic than the distant nodes. Furthermore, the presence of the jammer node close to these nodes can cause bottlenecks and more collisions. However, the MPH protocol has an average of retransmissions of 28% less than AODV and DSR, for random (*ρ* = 50, 80 packets/s) and reactive jamming. One possible explanation for this fact is that the AODV and DSR protocols affect more nodes under the most aggressive type of jamming (reactive), while in MPH, fewer nodes note the attack.

For the far case, we obtained that for a random jamming with *ρ* = 50 packets/s, we notice that MPH has 30% lower average retransmissions than AODV and DSR. With respect to a random jamming with *ρ* = 80 packets/s, MPH has 15% lower average retransmissions than AODV and DSR. With respect to reactive jamming, the average retransmissions values are still lower for MPH; for instance, the average for MPH is less than 13% against AODV and 15.4% with respect to DSR.

#### 6.1.2. CSMA Retries with Box and Whiskers Representation

**Figure 5:**
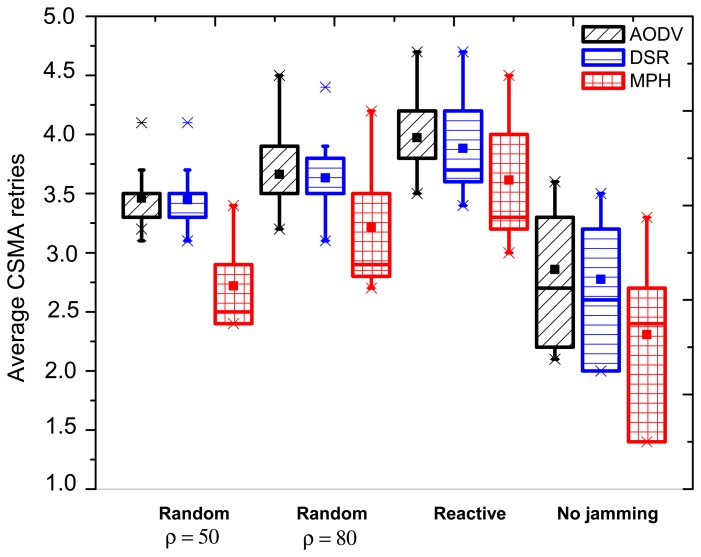
Average CSMA retries when the jammer node is in the middle of the topology.

The CSMA retries metric is presented in Figure 5. When there is no jamming, we can notice that the MPH protocol has an average of 2.3 of CSMA retries, while AODV has a mean of 2.86 and DSR 2.76. In addition, the MPH box shows that more than 50% of the nodes have average values between 1.38 and 2.7 (interquartile value), while AODV keeps 50% of the nodes between 2.2 and 3.3 average CSMA retries and DSR between two and 3.21. With these values, we can realize that MPH has fewer average CSMA retries and a better performance compared to the other two protocols.

Figure 5 exposes the types of jamming when the jammer node is in the middle of the topology. This jammer node location may affect many nodes, but its impact in the total delivery packet ratio should not be as strong as it is when the attacker node is closest to the destination node. With respect to the random jamming (*ρ* = 50 packets/s), AODV and DSR have an interquartile data distribution between 3.3 and 3.5 of the CSMA retry average values. This means that the nodes next to the coordinator deal with a more busy wireless medium due to the higher traffic load compared to distant nodes; therefore, the values in the CSMA retry parameter is greater than in other network nodes. Besides, if we consider that the central part of the topology is being affected by an attacker node, then almost all nodes in the network will have similar values regarding the evaluated variable. This also reveals that AODV and DSR have more affected nodes than MPH. This is because, for MPH, the box is slightly elongated, with an interquartile between 2.4 and 2.9. Furthermore, these values are significantly lower than in the case of AODV and DSR. For cases of random (*ρ* = 80 packets/s) and reactive jamming, the behavior is similar to the described jamming above, where MPH has lower values for the evaluated variable, and elongated boxes show that the affected nodes by the jammer are fewer than in the AODV and DSR cases.

Regarding the case when the jammer node is close to the coordinator and considering conditions of random jamming with *ρ* = 50 packets/s, MPH has 18.4% lower average CSMA retries compared to DSR and AODV. For a random jamming with *ρ* = 80 packets/s, MPH has 7.8% lower average CSMA retries. Finally, for reactive jamming, MPH is better by 8.8% than DSR and by 16.4% than AODV.

For the case when the jammer node is far from the coordinator node, we note that for random jamming with *ρ* = 50 packets/s, MPH has 40% less average CSMA retries with respect to the results for AODV and DSR. For a random jamming with *ρ* = 80 packets/s, MPH has 50% less average CSMA retries with respect to AODV and DSR. Finally, in the reactive jamming, MPH has 61% less average CSMA retries than the values obtained for AODV and DSR. This can be explained considering that the MPH protocol is not strongly affected when the attacker node is away from the destination. Although the average values of CSMA retries increase, MPH is able to encapsulate the attack, affecting only a few nodes. AODV and DSR tend to affect more nodes, and these values represent an increment in the average of CSMA retries in the nodes nearby the coordinator.

#### 6.1.3. Tries with Box and Whiskers Representation

**Figure 6:**
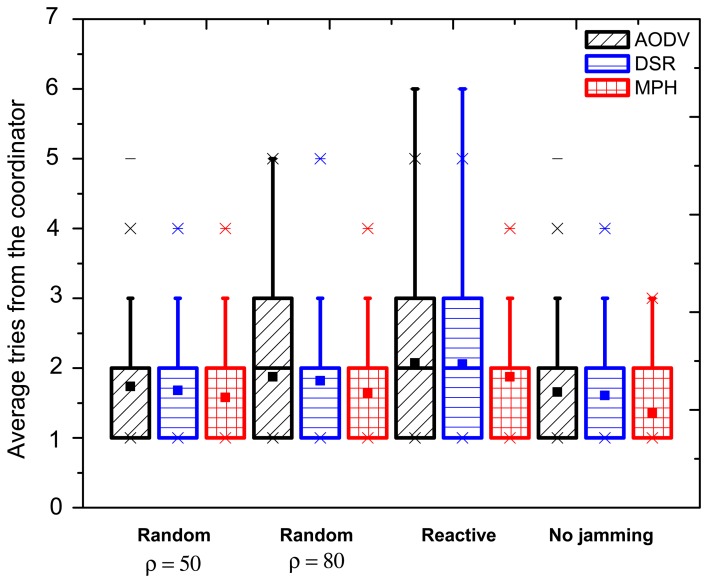
Average tries when the jammer node is in the middle of the topology.

In Figure 6, we display the average number of tries performed by the coordinator node to communicate with each node in the network. This variable indicates how much knowledge the coordinator node has about the network topology and the quality of the routes. When there is no jamming, the protocols have boxes of the same size for the three cases, which means that 50% of the data is distributed in approximately the same average values. However, the average value of tries for MPH is 1.33, whereas for DSR, it is 1.58 and for AODV 1.65. This means that MPH has 15.8% better performance connectivity of nodes with respect to DSR and 19.4% compared with AODV. Then, 50% of the nodes has between one and two tries, 25% of the nodes three tries and 25% of the nodes four tries for DSR. Besides, 50% of the nodes has between one and two tries, 25% of the nodes three tries, 24% of nodes four tries and 1% of the nodes five tries for AODV. While for MPH, 50% of the nodes has between one and two tries and 50% of nodes three tries (three being the maximum value reached in MPH under normal network behavior).

Figure 6 denotes metric tries when the jammer node is in the middle of the topology. We notice that in the random (*ρ* = 50 packets/s) jamming, the boxes of the three protocols remain the same as when there is no jamming (this is equivalent to 50% of nodes). However, for AODV, some nodes manifest variations on the evaluated parameter, which is 24% of nodes having four tries and 1% of nodes having five tries. For DSR and MPH, 50% of the remaining nodes presents four tries. However, for MPH, the average value of tries is 1.57, while for DSR, it is 1.7 tries. For random (*ρ* = 80 packets/s) jamming, AODV changes, and now, 50% of the nodes is between one and three tries and 50% of nodes four or five tries. DSR maintains 50% of nodes between one and two tries, another 25% of nodes three tries and the other 25% between four and five tries. MPH's percentages of nodes are maintained the same as in the random (*ρ* = 50 packets/s) jamming. Concerning the reactive jamming, AODV and DSR have 50% of nodes from one to three tries, and the other 50% of nodes has four, five and six tries. MPH maintains its previous features only with a small variation in the mean value of tries. With these numbers, we remark that the MPH protocol maintains its good performance and is not prone to collapse due to faulty links.

With respect to the case when the jammer node is close to the coordinator, for the random (*ρ* = 50 packets/s) and random (*ρ* = 80 packets/s) jammings, MPH has 12% low average tries with respect to AODV and 10% against DSR. One notable change can be observed in the reactive jamming, where AODV and DSR vary from one to three tries for 50% of the nodes. AODV varies between four and five tries for the other 50% of nodes, and for DSR, the remaining 50% of nodes has four tries. MPH maintains 50% of nodes varying between one and two tries, 24% of nodes three tries and only 1% of nodes four tries. This result shows the strength of the MPH against an attack and its better performance regarding encapsulation of failures.

For the case when the jammer node is far from the coordinator node, for the random (*ρ* = 50 packets/s) jamming, the three protocols have 50% of nodes with values between one and two tries and 25% of nodes three tries. For AODV, the remaining 25% of nodes has five tries, and the tries' average value is 1.8. In DSR case, the remaining 25% of nodes has five tries, and the tries mean value is 1.72. In contrast, for MPH, the remaining 25% of nodes has four tries, and the tries mean value is 1.6 (the lowest value compared with the other protocols). In the random (*ρ* = 80 packets/s) jamming, AODV has 50% of nodes between one and three tries, while DSR and MPH remain with the same values in the random (*ρ* = 50 packets/s) jamming case. For AODV, the remaining 25% of nodes has five tries, and the tries' average value increases to 1.95. In the DSR case, the remaining 25% has five tries, and the tries' average value increases to 1.87. In contrast, for MPH, another 24% of nodes has four tries and the remaining 1% five tries, and the tries' average value is 1.76. Finally, in the reactive jamming case, AODV and DSR have 50% of nodes between one and three tries, while MPH values remain unchanged (between one and two tries). The AODV and DSR cases have a very similar behavior, where the remaining 25% of nodes has five tries, and the tries' average value increases to 1.97. On the other hand, for MPH, the remaining 25% of nodes has four tries, and the tries' average value is 1.82. This MPH behavior describes that although the random (*ρ* = 80 packets/s) and reactive jammings affect MPH nodes similarly, MPH exposes better performance and link connectivity than AODV and DSR protocols.

### 6.2. Analysis Spatial Distribution of Performance Metrics

In this part, we show the 2D spatial representation of the studied performance metrics and exhibit the number of reached nodes by the coordinator in order to expose the self-configuration capacity of each protocol.

#### 6.2.1. Retransmissions with 2D Representation

**Figure 7:**
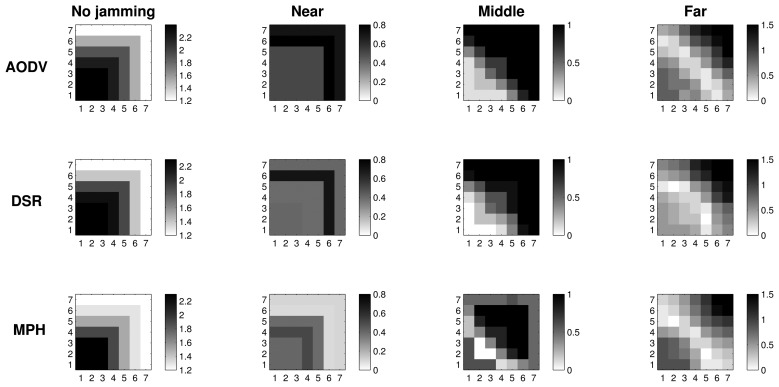
2D representation of the average retransmissions in the no jamming case and the difference between the values in the reactive and no jamming cases for each of the positions of the jammer node.

**Figure 8:**
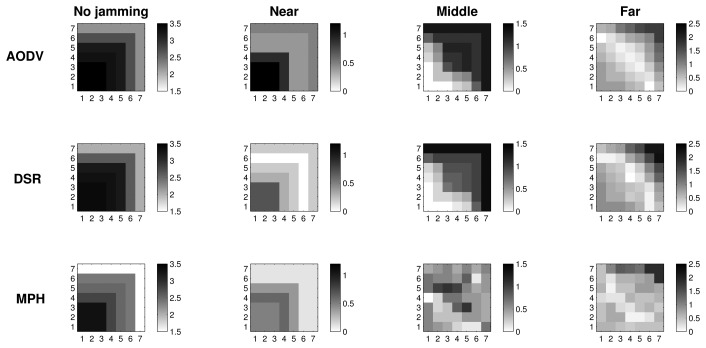
2D representation of the average CSMA retries in the no jamming case and the difference between the values in the reactive and no jamming cases for each of the positions of the jammer node.

**Figure 9:**
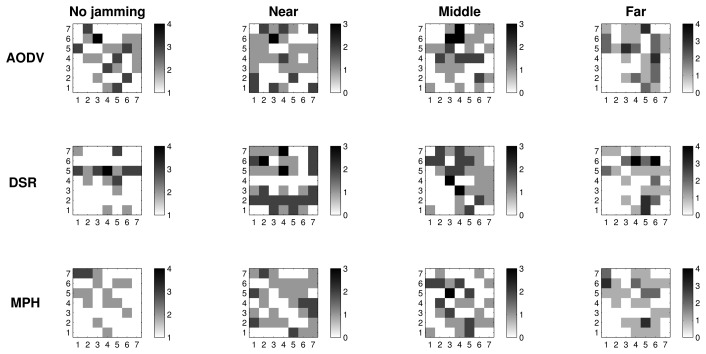
2D representation of the average tries in the no jamming case and the difference between the values in the reactive and no jamming cases for each of the positions of the jammer node.

Figures 7–9 present the worst case of jamming, the reactive case, for the three different positions of the jammer node. Each figure shows a grid of nodes that represents the used topology, a matrix of 7 × 7 nodes. Through a grayscale, we exemplify the maximum (close to black color) and minimum (close to white color) values. Grids of the “no jamming” column show the normal behavior of the network under no attack for each of the studied variables. The other three columns in each graph show the reactive case when the jammer node is close to (near column), when it is in the middle of the topology (middle column) and when it is far (far column) from the coordinator node. These grids have the change in the reactive jamming for each of the nodes, *i.e.*, the difference between the reactive jamming for each case and the value when there is no jamming for each of the nodes.

In Figure 7, we note that in the no jamming column, we have the average number of retransmissions for the AODV, DSR and MPH protocols. In these grids, we notice that there is a greater amount of retransmissions in nodes near the coordinator, because these nodes generate and forward traffic from other nodes. For this reason, collisions are more likely to occur and, hence, packet retransmissions. However, the MPH protocol has lower average retransmissions, and the nodes that are affected by collisions are less than AODV and DSR cases. Concerning the near column, we observe the difference in values between the reactive and no jamming case for each node. We note that for AODV, the biggest change is in the farthest nodes to the coordinator, because the jammer node is close to the coordinator, and this prevents the final communication, making the number of affected nodes greater in the network. Then, this attack position alters nodes that are far from the destination for AODV. Therefore, nodes close to the coordinator node have a change between 0.4 and 0.6, which shows that they are also significantly affected by the attack. For DSR, the attack is a little less aggressive, but also affects one of the farthest nodes crowns with respect to the coordinator with a change of about 0.6. The jamming has the lowest impact in terms of the number of nodes for the MPH; the change for nodes that are closer to the coordinator is approximately 0.4. Distant nodes have a change between 0.1 and 0.2. Regarding the middle column, AODV and DSR modified approximately one half of the nodes in the topology, while MPH encapsulates the attack slightly more with less affected nodes. With regard to the far column, AODV and DSR for the jammer node have more impact with a change of 1.5 in the near node jammer nodes. The MPH protocol has a change of one in about three nodes directly connected to the jammer node, and then, the change tapers to one, *i.e.*, MPH does not allow the attack to spread to multiple nodes as AODV and DSR.

#### 6.2.2. CSMA Retries with 2D Representation

Figure 8 shows the average CSMA retries when there is no jamming and reactive jamming cases when the jammer node is near, in the middle and far with regard to the coordinator. Note that the no jamming column for AODV has a maximum of 3.5 average CSMA retries for the closest nodes to the coordinator, while DSR has an intermediate value between three and 3.5, and MPH a value of three. We remark that as nodes move away from the coordinator, the CSMA retries are fewer, because these nodes forward less traffic, and therefore, the channel is less congested for communication. MPH reaches a value of 1.5 CSMA retries to the farthest nodes from the coordinator, while DSR has a value of two and AODV a value between two and 2.5. In the near column, we clearly observe that AODV has the highest difference (about one) for the nearest nodes to the coordinator. DSR continues with a difference value between 0.5 and one for these nodes and, finally, MPH with a value of 0.5. We note that MPH has the smallest change for the farthest nodes from the coordinator, showing that collisions caused by the jammer node do not affect a more extensive area in a large scale, as in the AODV and DSR cases. With respect to the middle column, the biggest change is observed for the AODV protocol, with values between one and 1.5 for about half of the nodes (the farthest part from the coordinator). DSR has a slight change between 0.5 and one for about the same half of nodes in the topology. While MPH has no abrupt changes in network nodes, it has only a few nodes with values between 0.5 and one around the central part of the topology (just around the jammer node position). The far column presents a similar behavior for the three protocols; however, MPH is the one with the least change (values between 1.5 and two) in the closest nodes with respect to the jammer node, and it is important to remark that although AODV presents the same values, MPH covers fewer nodes than AODV with these values.

#### 6.2.3. Tries with 2D Representation

Figure 9 exhibits the tries metric when there is no jamming in the network and reactive cases where the jammer node position is near, in the middle and far with respect to the coordinator. In the no jamming column, we observe that for all three protocols, nodes that are close to the coordinator needed fewer tries to be reached (one try). AODV and DSR reach up to four tries, while the maximum tries for MPH is three. For reactive jamming, cases in the other columns are not well defined in any of the protocols with respect to a specific pattern; however, MPH presents shaded nodes with lighter colors than the AODV and DSR cases, representing that the differences in the tries metric under reactive attack are softer than in the AODV and DSR protocols.

Table 3 helps us to visualize and quantify more easily the results that we observed in Figures 7, 8 & 9. In this table, we can perceive the three studied metrics (retransmissions, CSMA retries and tries). Numerical values represent the total sum of the difference values in each of the nodes for each jammer node position. These values show what protocol presents the biggest changes for the worst case (reactive jamming) better. We note that in most cases, the MPH has the smallest sum, meaning that the protocol is less prone to change when a reactive jamming exists in the network and that nodes are able to diminish the attack or focus it in a small region of the network.

#### 6.2.4. Retransmissions, CSMA Retries and Tries with 2D Representation for 225 Nodes

**Figure 10:**
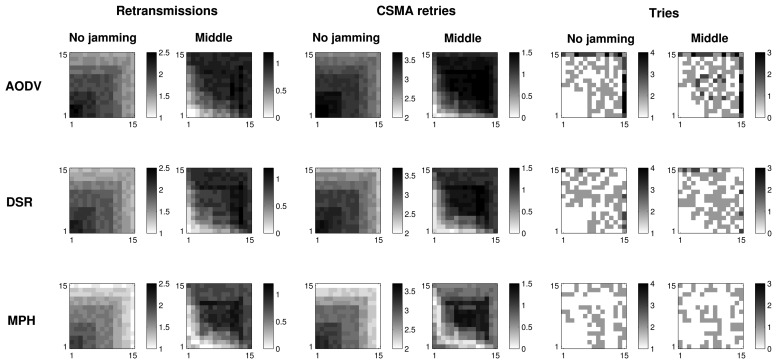
2D representation of the average retransmissions, CSMA retries and tries in the no jamming case and the difference between the values in the reactive and no jamming cases when the jammer node is in the middle of the topology.

In order to check the validity of our results and the behavior of the protocols, we simulated a network with more nodes. Figure 10 shows the results for a grid of 225 nodes. Retransmissions, CSMA retries and tries are simulated: two columns for each of these studied metrics. The left column in each case represents the case when there is no jamming, and the right column shows the difference between the no jamming case and the reactive jamming case when the position of the jammer node is at the center of the topology. These results are presented for the three protocols: AODV, DSR and MPH. The results expose that the performance of the three protocols tends to be similar when the number of nodes increases, so we can confirm that our grid of 49 nodes really explains what happens in the network. The AODV protocol presents the greatest average number of retransmissions, CSMA retries and tries. The MPH protocol offers the best resistance to the attack and causes fewer nodes to be affected. These results can be quantified in Table 4 to specifically observe the exact total numbers.

#### 6.2.5. Number of Reached Nodes by the Coordinator

Tables 5–7 show the number of reachable nodes by the coordinator for each of the positions of the jammer node. The column designated by the time, in seconds, presents periods of 10 s where there is an average count of how many nodes were reachable by the coordinator. The shaded rows in the tables show the times in which the attack is presented as random (*ρ* = 50 packets/s), random (*ρ* = 80 packets/s) or reactive case. As we can see, the position when the jammer node is in the middle of the topology is the one that most affects the total number of nodes that responded to the coordinator. This phenomenon may be caused due to the fact that when the jammer node being in the middle, interfering communication, it has complete circles of nodes around itself and may affect more of them. Whereas the jammer node is in one of the corners of the topology (near and far positions), it has incomplete circles of nodes; however, the far position affects fewer nodes than the nearest position. This may occur because an attack is stronger in a region with a high traffic concentration, such as the area near the coordinator node, because these nodes generate traffic and also forward traffic from other nodes to the coordinator node (destination). This causes a larger amount of collisions, and this can be increased if there is an attack present.

#### 6.2.6. Energy

**Figure 11:**
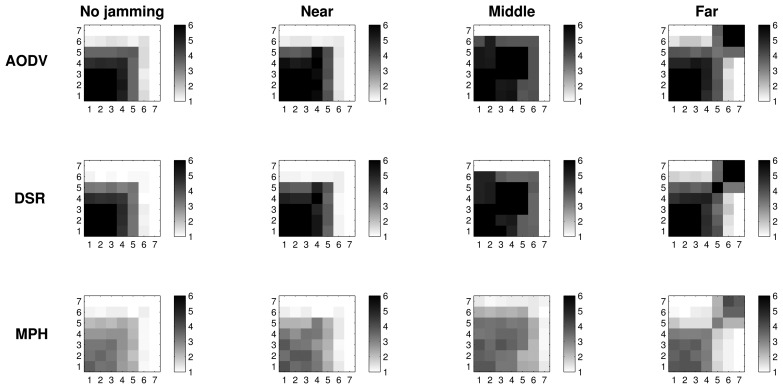
2D representation of energy for the no jamming and reactive cases when the jammer node is near to, in the middle of and far from the coordinator.

In Figure 11, the total energy at each node is shown in the case of reactive jamming. This is analyzed for the three positions of the jammer node: near, in the middle of the topology and far from the coordinator. The model of the energy is shown in Table 8, used for the Texas Instruments CC2530 chip. We can see that when there is jamming, the concentration of power is in the next coordinator nodes, because these nodes generate traffic and also carry traffic from other network nodes. However, the MPH protocol saves about 46% of the energy of these nodes closest to the coordinator. Furthermore, when we analyzed the total energy for the case when there was no jamming, the MPH protocol represents a 42% energy savings compared to AODV and 37% compared to DSR. Using Table 9, we have the quantization of the total energy for each of the protocols in each of the positions of the jammer node. We can see that when the jammer node is in the center of the attack, the topology appears to be more aggressive with respect to energy consumption, that is at the cost of a greater number of nodes. We note that the MPH protocol consumes less energy in all cases regarding AODV and DSR. For the close position, MPH has a savings of 44% compared to 41% AODV and DSR. For the center position, MPH has a savings of 41% compared to 38% AODV and DSR. For the far position, MPH has a savings of 43% compared to 42% AODV and DSR.

#### 6.2.7. Impacts of Different Attack Strategies on the Routing Performance

Table 10 reflects the impact of different attack strategies on the routing performance. The metrics studied throughout this work show the performance of each routing protocol (AODV, DSR and MPH). This table summarizes the positive (↑) or negative (↓) impact of better or worse performance on MPH against AODV and DSR. Here, we observe the types of studied jamming and their influence on each of performance metrics. This gives us an idea of what type of jamming is more aggressive in a network when the jammer is located around the center of the topology and the coordinator node (final destination) is at the corner.

## 7. Different Spatial Node Distributions

In this section, the main parameters of MAC and network layers for simulations of three different arrangements of nodes are described. Table 11 exhibits the parameters to simulate the three mentioned scenarios.

These scenarios depend on the location of the coordinator node and the jammer node. In the first configuration, when the coordinator node is located at one corner of the topology, every node in the network forwards and sends traffic to the coordinator. Routes are made longer here, and there is interference in the middle of the topology (jammer node). In the second configuration, the non-uniform random distribution, the coordinator node is located in the center of the topology, and the remaining nodes are distributed along the area. Sixty percent of the nodes, including the jammer node, are located near the central part of the topology, and the rest of the nodes, 40%, are distributed in the remaining area. In the third configuration, all nodes, the jammer and the coordinator are uniformly distributed. Analyzed metrics are tested under reactive jamming. These information and performance metrics are shown in Tables 12–14. The metrics shown here are those that have been working along the paper. These metrics are retransmissions, CSMA retries, tries from the coordinator, achievable nodes by the coordinator and energy. The measurements were made per node, *i.e.*, data reported in the tables are average numbers per node in 100 s of simulation time. Results exhibit that the MPH protocol has a better performance in all metrics with respect to the other three protocols.

The MPH protocol contribution is clearly seen in this comparison of performance metrics under the jamming attack. The three described scenarios help to visualize the impact of a jammer node with different node configurations better. A concrete contribution of the MPH protocol is self-configuration (this feature is demonstrated by achievable nodes by the coordinator), which helps to create links rapidly that were broken and to avoid packet loss (this feature is demonstrated by retransmissions and CSMA retries). Besides, we obtain better performance with low energy consumption, as we can observe in the energy metric for the three scenarios.

### 7.1. A Grid Where the Coordinator Is Located in One Corner and the Jammer is in the Middle

Table 12 shows the results for a grid of 49 nodes where the coordinator is located in a corner of the grid and all nodes generate and send traffic to it.

Regarding the retransmissions, MPH has 18.7% less retransmissions per node than AODV, and it has 16.3% less than DSR. With regard to the CSMA retries, MPH shows an average reduction of 11.4% against AODV and 9.4% against DSR. Concerning the tries, MPH has 14.3% less tries than AODV and 12.8% compared to DSR. Regarding the achievable nodes by the coordinator, MPH is the most self-configuring protocol, because it has 13.6% more achievable nodes known by the coordinator than AODV and DSR. Finally, with regard to energy, MPH has energy savings of 15.5% compared with AODV and 12.7% against DSR.

### 7.2. Non-Uniform Random Distribution

Table 13 exposes the results for a grid of 49 nodes where the coordinator is located in the center of the topology and all nodes generate and send traffic to it. The coordinator node is the destination. Moreover, we randomly localize 60% of nodes near the central part of the topology, including the jammer node, and the remaining nodes (40%) are distributed along the whole area in a random way.

Regarding the retransmissions, MPH has 16.8% less retransmissions per node than AODV and 12.9% less than DSR. With regard to the CSMA retries, MPH exhibits an average reduction of 13.6% against AODV and 11.4% against DSR. Concerning the tries by the coordinator, MPH is 30.2% less than AODV and 27.5% compared to DSR; this metric shows that the coordinator knows almost all routes to each node for the MPH protocol. Regarding achievable nodes by the coordinator, MPH has 16.7% more achievable nodes than AODV and 14.3% more than DSR. Finally, with regard to energy, MPH has energy savings of 16.7% compared with AODV and 11.2% against DSR.

The results of this scenario show that this configuration presents the worst case. This may be due to a high density of nodes being in the area near the coordinator node, and there is also the jammer node. This interfering node causes increased number of collisions and may actually cause more breakage of links because more nodes are affected. Because of the coordinator being the final destination, the packet loss is more affected by the presence of the jammer node.

### 7.3. Uniform Random Distribution

Table 14 manifests the results for 49 nodes under random positions along the area. All nodes generate and send traffic to the coordinator. Regarding the retransmissions, MPH has 19.4% less retransmissions than AODV and 18.1% less than DSR. With regard to the CSMA retries, MPH presents an average reduction of 17% against AODV and 12.3% against DSR. Concerning the tries, MPH exhibits 16% less tries by the coordinator than AODV and 13.9% less than DSR. Regarding achievable nodes by the coordinator, MPH has greater self-configuration features, such as 15.2% more achievable nodes than AODV and DSR. Finally, with regard to energy, MPH has energy savings of 18.4% compared with AODV and 16.6% against DSR.

Table 15 summarizes the percentages of MPH protocol performance against AODV and DSR. This table is intended to explain the contribution of MPH in each of the metrics studied in this work. The MPH protocol remarkably out-performs with respect to AODV and DSR; this may be due to its proactive-reactive characteristics, where its mechanisms for updating shorter routes and neighbor tables with parent-child relationships provide better versatility and speedier connection configuration and topology recovery. Moreover, MPH presents greater savings in energy consumption due to the optimization of routes and packet delivery times.

## 8. Conclusions

In this work, we have compared a recently proposed protocol, the multi-parent hierarchical (MPH) protocol, with two well-known protocols, the *ad hoc* on-demand distance vector (AODV) and the dynamic source routing protocol (DSR). In the simulation results, we observed that the MPH outperforms the other ones, for all metrics that we tested: the number of packet retransmissions, the CSMA inner loop retries, the number of nodes answering the queries from the coordinator (sink) node and the energy consumption.

The assessed metrics describe the network performance against various types of jamming. The large deviations of the metric variables for AODV and DSR when attacking conditions occur may make a later construction of a jamming detector easier. In contrast, for MPH, the changes in the metrics values are not as large as the deviations shown in AODV and DSR; this could be a desirable behavior from a performance perspective, but making the construction of a new detector more difficult; thus, further metrics could be proposed and analyzed according to specific protocols.

2D representation of the metrics distribution clearly shows that the MPH protocol can better manage the jamming impact on the network. We observed that under a jamming attack, MPH intrinsically isolates the area under attack and re-routes the traffic using the rest of the network; this is true, even if the attacker keeps moving. Therefore, WSNs with the MPH routing protocol present less performance deterioration when facing jamming activity. In MPH, the periodic updating of neighbor tables produces a positive effect to deal with the links affected by a jammer node. In addition, the combination of proactive and reactive mechanisms of MPH allow the reconfiguration of the network topology without generating excessive overhead.

Regarding AODV and DSR, which are reactive protocols, if the topology is constantly changing, the overhead increases due to the route discovery mechanism; this can generate more packet loss, and the attack becomes more aggressive and extensive, as can be seen in Figures 7–9.

From the simulation results, we can conclude that the resilience provided by routing protocols for self-configuring WSNs can help to deal with jamming activity. The network topology should adapt dynamically according to the available resources and the internal mechanisms of the underlying routing protocol. The resultant performance depends not only on the time to react against the attack, but also on the efficiency to achieve a new topology.

As future work, we suggest improving the reaction under a jamming attack. Since, at present, the MPH protocol uses a persistence counter to score neighbor steadiness, the use of a larger history of the neighbor activity may reduce the number of false positives in the decision to remove a node from the neighbor tables; however, this would produce an increased delay in the reconfiguration of the topology. Therefore, by helping this mechanism with the strength of the signal and other MAC and network layer parameters, a multi-feature detector or a routing protocol with better capabilities to deal with the jamming attacks could be constructed.

## Figures and Tables

**Table 1:** Comparative study of jamming: random and reactive.

**Analyzed Types of Jamming**	

**Random**	**Reactive**
Proactive	Reactive
Simple	More difficult to detect
Higher energy consumption	Lower energy consumption
Active mode (no idle periods)	Active mode (no idle periods)
Detection with signal strength mechanisms	More complex mechanisms for detection
Transmission packet rate = 50 and 80 packets/s	When a node detects activity, it transmits

**Table 2:** Simulation parameters. CSMA, carrier sense multiple access.

**Parameter**	**Value**
**Physical layer parameters**	

Sensitivity thresholds	−78 to −94 dBm
Transmission power	0 dBm

**MAC layer parameters**	

Maximum retransmission number	3
Maximum retry number	5
Maximum number of tries to reach a node from the coordinator	9
Packet error rate	1%–4%
Average frame length	22 bytes
Maximum number of backoffs	4
MAC protocol	IEEE 802.15.4
MAC layer	CSMA/CA

**Network layer parameters**	

Number of nodes	49
Discovery neighbor time	10 s
Maximum data rate	250 kbps
Scenario	Static nodes

**Table 3:** Sum of the values of differences for Figures 7–9.

**Retransmissions**			

	**Near**	**Middle**	**Far**
**AODV**	30.4	30.7	32.6
**DSR**	22.9	36	31.5
**MPH**	13.1	31.4	28.6

**CSMA Retries**			

	**Near**	**Middle**	**Far**

**AODV**	32.5	42.7	31.5
**DSR**	12.8	39	36.9
**MPH**	14.7	25	31.1

**Tries**			

	**Near**	**Middle**	**Far**

**AODV**	42	39	41
**DSR**	53	43	38
**MPH**	37	34	34

**Table 4:** Sum of the values of differences for Figure 10.

**Position of the Jammer Node = Middle**			

	**Retransmissions**	**CSMA Retries**	**Tries**
**AODV**	186.2	265.2	159
**DSR**	175.9	227.4	102
**MPH**	152.1	173.1	83

**Table 5:** Number of reached nodes by the coordinator when the jammer node is near.

**Number of Reached Nodes**									

**Time**	**Random** (*ρ* = 50 **packets/s**)			**Random** (*ρ* = 80 **packets/s**)			**Reactive**		

	**AODV**	**DSR**	**MPH**	**AODV**	**DSR**	**MPH**	**AODV**	**DSR**	**MPH**
10	43	44	44	44	43	44	44	45	46
20	47	48	48	48	47	47	47	48	48
30	47	48	48	48	47	48	48	48	48
40	46	46	46	47	45	46	46	46	47
50	41	43	46	41	40	44	39	40	44
60	41	43	46	41	40	44	39	40	44
70	41	43	45	40	41	45	42	41	45
80	46	47	48	47	47	46	44	45	46
90	47	47	48	48	47	48	46	46	48
100	48	48	48	48	48	48	47	47	48

**Table 6:** Number of reached nodes by the coordinator when the jammer node is in the middle of the topology.

**Number of Reached Nodes**									

**Time**	**Random** (*ρ* = 50 **packets/s**)			**Random** (*ρ* = 80 **packets/s**)			**Reactive**		

	**AODV**	**DSR**	**MPH**	**AODV**	**DSR**	**MPH**	**AODV**	**DSR**	**MPH**
10	44	44	44	43	43	45	44	45	46
20	47	47	48	48	47	47	47	48	48
30	47	48	48	48	47	48	48	48	48
40	44	44	46	43	43	46	41	41	45
50	40	40	45	39	38	44	38	38	44
60	40	40	45	39	38	44	38	38	44
70	41	42	45	40	40	44	40	41	45
80	45	47	48	47	47	46	44	45	46
90	47	47	48	47	47	48	46	46	48
100	47	48	48	48	48	48	47	47	48

**Table 7:** Number of reached nodes by the coordinator when the jammer node is far.

**Number of Reached Nodes**									

**Time**	**Random** (*ρ* = 50 **packets/s**)			**Random** (*ρ* = 80 **packets/s**)			**Reactive**		

	**AODV**	**DSR**	**MPH**	**AODV**	**DSR**	**MPH**	**AODV**	**DSR**	**MPH**
10	44	44	45	44	43	45	44	45	46
20	47	48	48	48	47	48	47	48	48
30	47	48	48	47	47	48	48	48	48
40	46	46	47	47	45	47	46	46	47
50	43	43	46	41	41	45	39	40	45
60	43	43	46	41	41	45	39	40	45
70	44	44	47	43	43	47	42	42	47
80	46	47	48	47	47	47	44	45	47
90	47	47	48	48	47	48	46	47	48
100	48	48	48	48	48	48	47	48	48

**Table 8:** Energy model [17].

	**Voltage (mV)**	**Current (mA)**	**Time (ms)**	**Energy (mJ)**
Start-up mode	120	12	0.2	0.288
MCUrunning on 32-MHz clock	75	7.5	1.7	0.956
CSMA/CA algorithm	270	27	1.068	7.78
Switch from RX to TX	140	14	0.2	0.392
Switch from TX to RX	250	25	0.2	1.25
Radio in RX mode (processing and waiting)	250	25	4.1915	26.2
Radio in TX mode	320	23	0.58	4.26
Shut down mode	75	7.5	2.5	1.41

**Table 9:** Sum of values of the total energy for Figure 11.

**Energy (J) for Reactive Jamming**				

	**No Jamming**	**Near**	**Middle**	**Far**
**AODV**	149.1372	160.8547	209.6946	191.1689
**DSR**	136.8437	151.0130	199.6251	188.6629
**MPH**	86.4993	89.4854	124.3036	109.1902

**Table 10:** Impact of the types of jamming when the jammer node is in the middle.

**Impact of the Types of Jamming of the MPH Protocol**			

	**Random *ρ*** = 50	**Random *ρ*** = 80	**Reactive**
**Retransmissions**			

**Against AODV**	1.6% ↑	2.0% ↓	2.2% ↓
**Against DSR**	1.2% ↑	9.4% ↑	12.8% ↑

**CSMA retries**			

**Against AODV**	5.6% ↑	25.3% ↑	41.4% ↑
**Against DSR**	4.8% ↑	22.6% ↑	35.9% ↑

**Tries**			

**Against AODV**	3.1% ↑	9.9% ↑	12.8% ↑
**Against DSR**	2.7% ↑	9.5% ↑	20.9% ↑

**Achievable nodes by the coordinator**			

**Against AODV**	11.1% ↑	11.4% ↑	13.8% ↑
**Against DSR**	11.1% ↑	13.6% ↑	13.8% ↑

**Energy**			

**Against AODV**	26.3% ↑	33.7% ↑	40.7% ↑
**Against DSR**	23.2% ↑	30.1% ↑	37.7% ↑

**Table 11:** Parameters for the tested scenarios.

**Parameter**	**Value**
Maximum data rate	250 kbps
Discovery packet time	10 s
Transmission power	0 dBm
Receiver sensitivity	−85 dBm
Average frame length	22 Bytes
MAC layer	CSMA/CA
Maximum number of backoffs	4
Packet loss	1%
Coverage radio	50 m
Mode	Active
Jamming	Reactive
Scenario	Static
Area	150 m × 150 m

**Table 12:** Scenario 1.

**Topology of Figure 3 Where the Coordinator is Located in One Corner**					

	**Retransmissions**	**CSMA Retries**	**Tries**	**Achievable Nodes by the Coordinator**	**Energy (J)**
**AODV**	2.72	4.03	2.23	38	4.39
**DSR**	2.64	3.94	2.19	38	4.25
**MPH**	2.21	3.57	1.91	44	3.71

**Table 13:** Scenario 2.

**Non-Uniform Random Distribution**					

	**Retransmissions**	**CSMA Retries**	**Tries**	**Achievable Nodes by the Coordinator**	**Energy (J)**
**AODV**	2.91	4.42	3.14	35	4.86
**DSR**	2.78	4.31	3.02	36	4.56
**MPH**	2.42	3.82	2.19	42	4.05

**Table 14:** Scenario 3.

**Uniform Random Distribution**					

	**Retransmissions**	**CSMA Retries**	**Tries**	**Achievable Nodes by the Coordinator**	**Energy (J)**
**AODV**	2.53	3.87	2.06	39	4.13
**DSR**	2.49	3.66	2.01	39	4.04
**MPH**	2.04	3.21	1.73	46	3.37

**Table 15:** Better performance of the MPH protocol against AODV and DSR.

**Percentage of Better Performance of the MPH Protocol**					

	**Retransmissions**	**CSMA Retries**	**Tries**	**Achievable Nodes by the Coordinator**	**Energy (J)**
**Scenario 1**					

**Against AODV**	18.7%	11.4%	14.3%	13.6%	15.5%
**Against DSR**	16.3%	9.4%	12.8%	13.6%	12.7%

**Scenario 2**					

**Against AODV**	16.8%	13.6%	30.2%	16.7%	16.7%
**Against DSR**	12.9%	11.4%	27.5%	14.3%	11.2%

**Scenario 3**					

**Against AODV**	19.4%	17%	16%	15.2%	18.4%
**Against DSR**	18.1%	12.3%	13.9%	15.2%	16.6%

## References

[1] Zanella A., Bui N., Castellani A., Vangelista L., Zorzi M. (2014). Internet of Things for Smart Cities. IEEE Internet Things J..

[2] Pelechrinis K., Iliofotou M., Krishnamurthy S. (2011). Denial of Service Attacks in Wireless Networks: The Case of Jammers. IEEE Commun. Surveys Tutor..

[3] Friginal J., de Andres D., Ruiz J., Martinez M. (2014). A survey of evaluation platforms for ad hoc routing protocols: A resilience perspective. Comput. Netw..

[4] Xu W., Ma K., Trappe W., Zhang Y. (2006). Jamming Sensor Networks: Attack and Defense Strategies. IEEE Netw..

[5] Del-Valle-Soto C., Mex-Perera C., Olmedo O., Orozco-Lugo A., Galván-Tejada G., Lara M. (2014). On the MAC/Network/Energy Performance Evaluation of Wireless Sensor Networks: Contrasting MPH, AODV, DSR and ZTR Routing Protocols. Sensors.

[6] Del-Valle-Soto C., Mex-Perera C., Olmedo O., Orozco-Lugo A., Galván-Tejada G., Lara M. An efficient Multi-Parent Hierarchical Routing Protocol for WSNs.

[7] Kiwior D., Lam L. Routing Protocol Performance over Intermittent Links.

[8] Mingyan L., Koutsopoulos I., Poovendran R. (2010). Optimal Jamming Attack Strategies and Network Defense Policies in Wireless Sensor Networks. IEEE Trans. Mob. Comput..

[9] Misra S., Singh R., Mohan S.V.R. (2011). Geomorphic zonalisation of wireless sensor networks based on prevalent jamming effects. IET Commun..

[10] Yu M., Su W., Kosinski J., Zhou M. A new approach to detect radio jammings in wireless networks.

[11] Jiang S., Xue Y. (2011). Providing survivability against jamming attack for multi-radio multi-channel wireless mesh networks. J. Netw. Comput. Appl..

[12] Awerbuch B., Richa A., Scheideler C. A jamming-resistant MAC protocol for single-hop wireless networks.

[13] Law Y.W., Palaniswami M., van Hoesel L., Doumen J., Hartel P., Havinga P. (2009). Energy-efficient link-layer jamming attacks against wireless sensor network MAC protocols. ACM Trans. Sens. Netw. (TOSN).

[14] Xu W., Trappe W., Zhang Y., Wood T. The feasibility of launching and detecting jamming attacks in wireless networks.

[15] Perkins C., Royer E. Ad-hoc on-demand distance vector routing.

[16] Maltz D., Broch J., Jetcheva J., Johnson D. (1999). The effects of on-demand behavior in routing protocols for multihop wireless ad hoc networks. IEEE J. Sel. Areas Commun..

[17] Texas Instruments www.ti.com/lit/an/swra292/swra292.pdf.

[18] Instruments, Texas (2011). A True System-on-Chip Solution for 2.4-GHz IEEE 802.15.4 and ZigBee Applications.

[19] (2007). Wireless Medium Access Control (MAC) and Physical Layer (PHY) Specifications for Low-Rate Wireless Personal Area Networks (WPANs).

[20] Montgomery D.C., Runger G.C. (2007). Applied Statistics and Probability for Engineers.

